# Certificates of Death

**Published:** 1862-07

**Authors:** 


### CERTIFICATES OF DEATH.

For a few months past, we notice, the Health Physician has appended to his report of deaths, *by whom certified;* and we are most thankful for this important item of intelligence, indeed we may say this most important item of this record.

Our report of deaths, as made by the undertakers and irregular practitioners is a foolish farce, and of no more value than a blank. To ask physicians to make correct and full death certificates, and yet accept those offered from all other sources, is an imposition and outrage, and we think it about time to regulate this matter, so if possible to prevent the further use of the death certificate, as a cover and safe-guard for the most revolting crime.

Our attention has recently been called to this regulation of our City, by being invited to visit in her dying moments, one of the victims of an Indian abortionist, where we learned that other victims had been buried from her institution, at very unseasonable hours, even in the night, unattended, unmourned, unwept,—the *Death Certificate*, by Madam Lachceil.

In visiting this institution, under the protection of the police, we had an opportunity of looking into, and going into, a more actual hell, than we had supposed could be found upon the face of the earth; no voice or language can give the slightest idea of it. It appeared a den of crime, murder, death, and all other damnable things, conceived or dreamed; and yet we find a certificate of death from the presiding spirit, is received by a sexton, without hesitation as representing truly the circumstances, causes and time of death.

In the month of May we notice only sixteen deaths are certified by irregular practitioners, with a total of one hundred and twenty-seven. This includes the certificates from Homœopathists, Thompsonians, Eclectics, Hypodermists, Clairvoyants, Electropathists, Indian Physicians, Laying on hand practitioners or Charm Doctors. By it, we learn that comparatively very few are really attended when they come to die, by quacks. That there should be any, so stupid and uninformed in the tricks and deceptions of the above named classes of imposters, as to be willing, at any time, to receive them to their houses and sick rooms; above all, to receive their attendance in the hour of death, is a mystery and a wonder.

Systems of absurdity, more barbaric and heathenish, more superstitious and irrational than throwing children in the Ganges, or worshipping wooden gods, are openly defended and advocated by people professing Christianity, living in good dwellings, wearing fashionable clothing, and associating in intelligent circles, and supposed by everybody to be able to read. Can we regard people thus blinded and foolish with respect, or meet them upon any ground of social equality, in the least desiring their acquaintance. God grant us grace to tolerate their acquaintance that we may have opportunity of pouring into their darkened understandings some of the first principles of common perception and common sense. While the community encourage and support these various forms of deception and pretension, we may look on, and observe the workings of a most gigantic cheat.

The pretension, brow-beating and bluster, of these irregulars in the practice of medicine, tends to keep down the indignation of mankind; otherwise to offer a certificate of death from one of them, would be regarded sufficient ground of suspicion, and a Coroner would be invited to investigate the cause of death. These returns would be liable to inaccuracies when made by well qualified physicians; the often obscure causes of death would not in all cases be discovered; but these reports when properly guarded, are capable of being made for the most part, reliable, and very valuable. To receive from all sources indiscriminately, these certificates, is so manifestly absurd, as to admit of no defence; and we hope to see this just ground of complaint speedily removed, by the energetic and united action of those having control.

